# Antenatal care services utilization during COVID-19 second wave attack in Pasuruan, Indonesia

**DOI:** 10.25122/jml-2021-0238

**Published:** 2022-01

**Authors:** Novida Ariani

**Affiliations:** 1. Midwifery Department, Faculty of Medicine, Brawijaya University, Malang, Indonesia

**Keywords:** antenatal care, maternal health service facilities, COVID-19 pandemic

## Abstract

Indonesia is currently experiencing the second wave of the COVID-19 pandemic, impacting maternal health services and maternal mortality. This research aims to investigate the use of antenatal care (ANC) during the COVID-19 second wave and the factors that play a role in this situation. A cross-sectional study was conducted during July 2021 on 344 pregnant women in primary, secondary, and tertiary maternal health care facilities in Pasuruan Regency, Indonesia. The data collection technique was simple random sampling, with face-to-face interviews assisted by questionnaires. Logistic regression and adjusted odds ratio with 95% CI and p<0.05 were performed to identify a significant relationship. 136 (39.5%) pregnant women did not use ANC services during the second wave of the COVID-19 outbreak. Husband’s support (AOR=13.814, 95% CI: 8.090–23.588), believing that pregnant women are not afraid of contracting COVID-19 (AOR=6.501, 95% CI: 3.904–10.825), easy access to transportation (AOR=12.145, 95% CI: 6.186–23.846), ease of ANC fees (AOR=4.105, 95% CI: 2.424–6.950), no lockdown policy (AOR=3.130, 95% CI: 1.983–4.940), knowledge regarding COVID-19 (AOR=2.975, 95% CI: 1.793–4.938), COVID-19 information on social media (AOR=3.035, 95% CI: 1.179–7.815), COVID-19 prevention protocols in health facilities (AOR=8.478, 95% CI: 3.611–19.903) were predictors of ANC utilization. This encourages the importance of prioritizing health services for pregnant women during the pandemic, overcoming the fear of contracting COVID-19 through maternal education, husband support, easy access to ANC, and improving the quality of ANC service facilities.

## Introduction

The World Health Organization (WHO) stated that COVID-19 became a global pandemic throughout the world in March 2020 [1]. Since that day, the pandemic has spread worldwide, including in Indonesia. This country is currently facing the second wave of the COVID-19 pandemic, estimated to be worse than the previous one [2]. The addition of daily cases reaches more than 50,000 cases per day [3]. This causes Indonesia to become the new epicenter of COVID-19 in Asia [4]. A report in Botswana stated that maternal health services were one of the services affected by the COVID-19 pandemic. It causes mothers to be afraid of being infected when visiting health care facilities, thereby reducing their visits. The existence of restrictions on mobilization during the pandemic also contributed to the low number of visits [5]. Access to maternal health services should prevent morbidity and mortality through early detection of abnormalities in pregnancy. Consequently, barriers to health services access delay diagnosis and treatment. In addition, the COVID-19 pandemic also causes anxiety and psychological disorders in pregnant women, which are associated with poor delivery outcomes [6]. COVID-19 has also been reported to increase maternal mortality [7]. A study in Brazil shows that maternal mortality increased by more than 13% during the COVID-19 pandemic. This increase could come from direct and indirect factors [8]. The role of indirect factors on the use of antenatal care still requires further investigation. There is still an information gap regarding this in Indonesia.

The pattern of antenatal care utilization during the COVID-19 pandemic is fundamental to acknowledge. Moreover, the COVID-19 second wave attack is still occurring in Indonesia. By acknowledging this gap, it will be possible to conclude a strategy to overcome the obstacles in maternal services during the COVID-19 pandemic, which seems to hint at no departure from Indonesia. This strategy is also necessary to deal with another wave of the COVID-19 pandemic given new mutation variants. This new strategy could include an appropriate education for pregnant women, easy access to maternal service facilities, perhaps even modification of health services through telemedicine or door-to-door visits by health workers to the pregnant women’s residence.

## Material and Methods

This cross-sectional research was conducted among pregnant women in July 2021, when lockdown occurred in Indonesia due to the COVID-19 second wave attack (called PPKM). This research was conducted in East Java, one of the most populous provinces in Indonesia and the highest contributor to the incidence of COVID-19 in Indonesia. Precisely in the Pasuruan Regency, a city that represents the description of cities in East Java. Subjects were included from primary, secondary, and tertiary maternal health service facilities to reflect the pattern of ANC utilization at various levels of maternal health facilities. Primary, secondary, and tertiary health facilities are interrelated in terms of tiered referrals. The total population of Pasuruan Regency is 1,637,682 people. There is one tertiary referral hospital, six secondary referral hospitals, and 33 primary health care centers [9].

The sample size was calculated using a single population proportion formula based on assumptions that included: 95% confidence interval, 5% margin of error, and the assumption of 70% antenatal care visits proportion because there were no previous studies. Hence, the total sample size was 329. The final sample size was determined at 345, considering the assumption of 5% non-response. The sampling technique was carried out from tertiary, secondary, and primary health facilities. Three health facilities were selected purposively. The number of samples taken at these health facilities was generated proportionally based on the average number of antenatal care services from the last 6 months. The research subjects in each health facility were selected by simple random sampling until the required sample size was reached. The independent variables of the research were socio-demographic, socio-cultural, socio-economic factors, midwifery, and health facilities. The dependent variable is ANC utilization, namely the use of antenatal care regarding the number of visits according to WHO standards and getting additional tablets of iron, folic acid, tetanus toxoid injections, pregnancy check-ups, and education. Accepting all ANC treatment was coded as yes, receiving partial or not receiving was coded as no. Data was collected using a structured questionnaire. Questionnaires were taken through interviews by health workers, namely: 7 midwives and a doctor. Midwives as data collection officers and doctors as supervisors to ensure data quality which includes completeness, clarity, and accuracy of data. Health workers were previously given 2 days of training to conduct data collection interviews. The questionnaires were taken from various literature and modified according to the research objectives. Social, demographic, economic, and cultural aspects, health services amid a pandemic, transportation, and government policies are included as the variables studied. Furthermore, a pre-test was carried out to see the feasibility of the interview results, including completeness, accuracy, and difficulties that could arise during the interview. A solution was found from the pre-test results if there were problems during the interview. Before the interview, interview officers used all infection prevention protocols, including medical face masks, social distancing, and hand sanitizers.

All data were checked for inconsistency, and incompleteness, then entered into Epi-data version 4.6 and analyzed using SPSS version 26. Descriptive statistics were described using frequencies, percentages, and tables. Hosmer Lemeshow fit test was performed. Bivariable and multivariable logistic regression was performed to identify a significant relationship between the independent variable and the dependent variable. Adjusted odds ratio (AOR) with 95% CI indicates the strength of the association. A p-value<0.05 was used in the logistic regression analysis to express statistical significance.

## Results

### Socio-economic and demographic characteristics of the research subjects

345 pregnant women were involved in this research, and 344 (99.7%) pregnant women responded to our questionnaire. Most of the subjects (52.9%) were in the 20–35 age group. 223 participants (64.8%) had a secondary school education, 198 subjects (57.6%) were housewives. 146 of the subjects’ houses (42.4%) were 5–10 km from health facilities. Only 27 subjects (7.8%) earned an average monthly income of more than 5 million Rupiah per month (Table 1).

**Table 1.** Socio-demographic characteristics of pregnant women attending antenatal care at public health facilities in Pasuruan, Indonesia during COVID-19 second-wave outbreak, 2021 (n=344).

**Variable**	**Category**	**Frequency**	**Percentage**
**Maternal Age**	<20 years old 20–35 years old >35 years old	126 182 36	36.6% 52.9% 10.5%
**Maternal Education**	Elementary School Secondary or High School Higher Education	49 223 72	14.2% 64.8% 21%
**Husband Education**	Elementary School Secondary or High School Higher Education	26 258 60	7.6% 75% 17.4%
**Maternal Occupation**	Unemployed Employed	198 146	57.6% 42.4%
**Husband Occupation**	Unemployed Employed	64 280	18.6% 81.4%
**Distance from Health Facility**	<5 km 5–10 km >10 km	126 146 72	36.6% 42.4% 21%
**Estimated Monthly Income**	<1 M 1–5 M >5 M	79 238 27	23% 69.2% 7.8%

### Obstetric characteristics of pregnant women

Among the 344 research subjects, 196 (57.2%) had 2^nd^ to 4^th^ pregnancies. Most of the 249 subjects (72.6%) had no bad obstetric history (BOH). Most of the 155 subjects (45%) were in the 3^rd^ trimester of pregnancy (Table 2).

**Table 2.** Obstetric characteristics of pregnant women attending antenatal care at public health facilities in Pasuruan, Indonesia during COVID-19 second-wave outbreak, 2021 (n=344).

**Variable**	**Category**	**Frequency**	**Percentage**
Bad Obstetric History	No Yes	249 95	72.6% 27.4%
Gravidity	Primigravida 2 s/d 4 5≥	141 196 6	41.1% 57.2% 1.7%
Gestational Age	1^st^ Trimester 2^nd^ Trimester 3^rd^ Trimester	68 121 155	19.8% 35.2% 45%

### The Influence of COVID-19 Pandemic on ANC

#### Service Utilization

**Figure 1.**
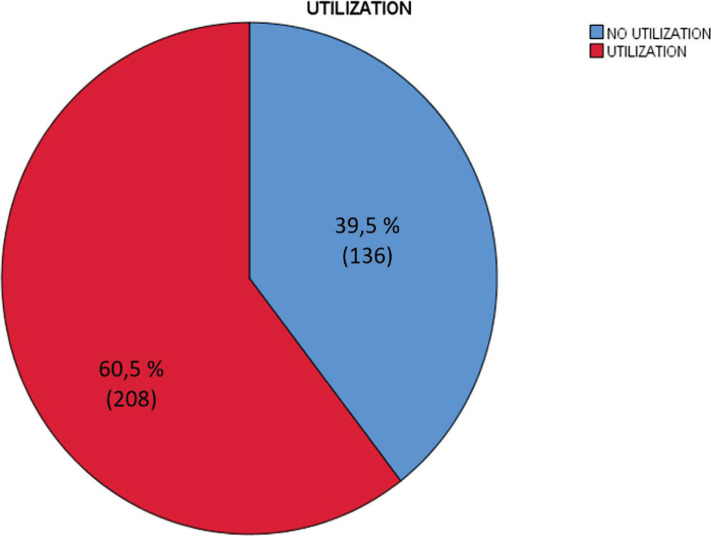
Service Utilization, n=344.

The research revealed that 136 (39.5%) subjects did not use antenatal care (ANC) (Figure 1) in an ideal maternal health facility, according to WHO. Subsequently, they did not receive the quantity and completeness of ANC services that should have been received, including folic acid, iron tablets, examination, tetanus toxoid injections, and education.

#### Problems of Maternal Health Service during COVID-19 Second Wave Pandemic Attack in Pasuruan, Indonesia

**Figure 2.**
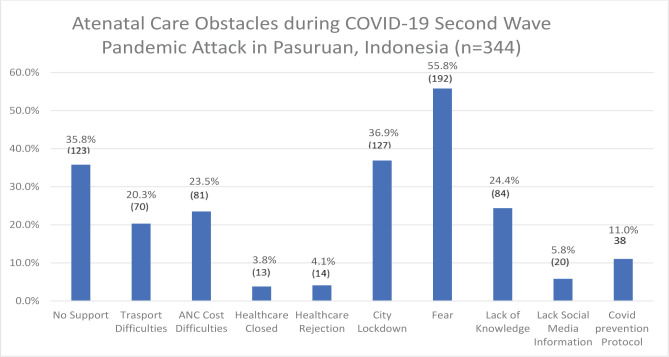
Service Utilization, n=344.

Various kinds of antenatal care obstacles were raised from the research subjects (Figure 2) during the COVID-19 second wave attack. Of these, 123 women (35.8%) stated that their husbands did not support them to access ANC. 70 (20.3%) pregnant women expressed difficulty getting transportation to health facilities to access ANC. 81 (23.5%) pregnant women experienced difficulty in ANC costs. 13 (3.8%) respondents admitted that health facilities were closed, and 14 (4.1%) respondents admitted that health facilities rejected them during the second wave attack. 127 (36.9%) people said they had difficulty with ANC due to the lockdown in their area. Most pregnant women 192 (55.8%) were afraid of contracting COVID-19 when accessing ANC. A total of 84 (24.4%) pregnant women admitted that they did not know much about COVID-19. As many as 20 (5.8%) pregnant women claimed to have never received information about COVID-19 from social media. Meanwhile, 31 (11.04%) pregnant women stated that good health protocols were not implemented in health facilities.

#### Factors related to antenatal care use

Various factors such as maternal age, mother’s occupation, husband’s occupation, mother’s education status, husband’s education status, distance from residence from health facilities, estimated monthly income, gravidity, husband’s support, health service closures, denial of health services, fear, lockdown policies influenced antenatal care use. Mothers’ knowledge regarding COVID-19 and social media information regarding COVID-19 were tested to assess the relationship with ANC access using bivariable logistic regression. The p-value<0.05 was used to determine the significance. From the test results, maternal age, husband’s occupation, the distance of residence from health facilities, husband’s support, difficulty in ANC costs, transportation difficulties, fear, lockdown policies, knowledge about COVID-19, information on social media about COVID-19, the implementation of the COVID-19 prevention protocol in health facilities is a predictor for accessing maternal health services (ANC).

The probability of ANC use during the COVID-19 pandemic among mothers aged 20–35 years was 3.200 times (CI 1.986–5.156) higher, while those over 35 years were 2.630 times (CI 1.208–5.726) higher than those aged less than 20 years. The distance of more than 10 km from the mother’s residence has a chance of 0.297 ANC utilization (CI 0.162–0.544) times lower. Husbands who work have an ANC probability of 2.314 (CI 1.334–4.015) times higher than husbands who do not work. A previous bad pregnancy history had 1.833 times higher ANC chance (CI 1.104–3.044) than those without a bad pregnancy history. Husband’s support, surprisingly, has an ANC probability of up to 13.814 (CI 8.090–23.588) times higher than those without husband’s support. The availability of transportation also has a higher chance of ANC up to 12.145 (CI 6.186–23.846) times. Pregnant women who can afford the costs that may arise during ANC have a 4.105 (CI 2.424–6.950) times higher chance than pregnant women who cannot afford ANC. The absence of a lockdown near the place of residence of pregnant women has a 3.130 (CI 1.983–4.940) times higher chance of utilizing maternal services. Pregnant women who are not afraid of COVID-19 have a 6.501 times higher chance of ANC (CI 3.904–10.825). In addition, the possibility of pregnant women who have sufficient knowledge about the transmission of COVID-19 has a higher chance of 2.975 (CI 1.793–4.938) times. Pregnant women who received sufficient information on social media about COVID-19 had a higher chance of maternal services 3.035 (CI 1.179–7.815) times. Implementation of COVID-19 prevention protocols in health care facilities has a chance of 8.478 (3.611–19.903) times higher than facilities which do not apply the protocol (Table 3).

**Table 3.** Multivariable logistic regression to assess factors associated with antenatal care utilization during COVID-19 second wave pandemic attack, in Pasuruan, Indonesia, 2021 (n=344).

**Variables**	**Utilization**		**Odd Ratio (95% CI)**	**p-value**
	**No**	**Yes**		
**Age Category**				
<20 years old 20–35 years old >35 years old	71 53 12	54 129 24	1 3.200 (1.986–5.156) 2.630 (1.208–5.726)	- 0.000 0.015
Distance				
<5 km 5–10 km >10 km	37 57 42	89 89 30	1 0.649 (0.391–1.078) 0.297 (0.162–0.544)	- 0.095 0.000
**Maternal Education**				
Elementary School Secondary and High School Higher Education	18 91 27	31 132 45	1 0.842 (0.444–1.596) 0.968 (0.456–2.052)	- 0.599 0.932
**Husband Education**				
Elementary School Secondary and High School Higher Education	13 102 21	13 156 39	1 1.529 (0.682–3.432) 1.857 (0.730–4.726)	- 0.303 0.194
**Maternal Occupation**				
Unemployed Employed	75 61	123 85	1 0.850 (0.549–1.315)	- 0.465
**Husband Occupation**				
Unemployed Employed	36 100	28 180	1 2.314 (1.334–4.015)	- 0.003
**Income**				
<1 M 1–5 M >5 M	35 90 11	44 148 16	1 1.308 (0.781–2.190) 1.157 (0.477–2.808)	- 0.307 0.747
**Gravidity**			1.042 (0.836–1.300)	0.713
**BOH**				
No Yes	108 28	141 67	1 1.833 (1.104–3.044)	- 0.019
**Transportation**				
Transport Difficulties No Difficulties	58 78	12 196	1 12.145 (6.186–23.846)	- 0.000
**ANC Cost**				
Cost Difficulties No cost Difficulties	53 83	28 180	1 4.105 (2.424–6.950)	- 0.000
**Healthcare Closed**				
Closed Open	9 127	5 203	1 2.877 (0.943–8.778)	- 0.063
**Healthcare Rejection**				
Yes No	13 123	0 208	1 2731861673 (0.000)	- 0.998
**Lockdown**				
Yes No	72 64	55 153	1 3.130 (1.983–4.940)	- 0.000
**Fear**				
Yes No	110 26	82 126	1 6.501 (3.904–10.825)	- 0.000
**Protocol**				
No Yes	31 105	7 201	1 8.478 (3.611–19.903)	- 0.000
**Knowledge**				
Not know Know	50 86	34 174	1 2.975 (1.793–4.938)	- 0.000
**Social Media**				
No information Socmed Information Socmed	13 123	7 201	1 3.035 (1.179–7.815)	- 0.021

## Discussion

This research showed that the prevalence of antenatal care use during the COVID-19 second wave attack was 60.5% 95% CI [61.5–68.0]. This prevalence is lower than the overall service utilization in Pasuruan Regency before the pandemic, which the local government claimed to be above 90%. This research indicates that ANC utilization is still higher than in northeast Ethiopia [10] but lower than central Ethiopia [11]. The low utilization of this service resulted from the COVID-19 second wave attack in Indonesia, which is worse than the first attack. The high prevalence of confirmed victims and those who died in the COVID-19 second wave attack may trigger fear in the community. Restrictions on mobility imposed by the government through lockdowns and transportation restrictions have caused layoffs in various regions. All of this may cause pressure on people’s lives, thus contributing to the decline in ANC utilization. This research indicates the need for strategic policies to overcome the low utilization of ANC during the second wave of pandemic attacks in Indonesia, or perhaps the next wave that may still occur. Strategic policies are needed to optimize telemedicine, health forces, and village midwives who operate door to door for ANC services during the pandemic wave.

Various socio-economic factors contributed to the low utilization of maternal services during the COVID-19 second wave attack, such as the age and occupation of the husband. Age over 20 years seems to increase the opportunity to use maternal services in accordance with a previous study from 31 countries [12]. According to previous studies, employed husbands also increase the chances of utilizing maternal services [13]. It seems that husbands who work can meet the costs incurred when seeking ANC services. The working status of mothers does not increase the chances of seeking ANC services, perhaps due to the strong culture that the husband’s income is used to meet household needs, including ANC services. However, increasing the amount of income does not increase the chances of seeking maternal services. This may be due to the very low cost of ANC services in Indonesia, and even low incomes of less than 1 million per month can pay for these. This research also shows that education does not increase the chances of seeking ANC services, in contrast to previous studies [14, 15]. This may be due to the fear of contracting COVID-19, which impacts pregnant women despite their high level of education.

According to previous studies, obstetric factors such as parity, a poor birth history, and greater gestational age also increase the chances of using ANC [16–18]. Lockdown reduces ANC utilization. These results are in line with previous research [10]. Of course, the lockdown limits the space for movement and mobilization of the population. In line with these results, transportation difficulties also reduce ANC utilization. Indonesia’s large geographical area requires the mobilization of pregnant women to reach maternal health services. However, public transportation is not allowed during the government’s lockdown policy, reducing ANC utilization if it is located far from home. It also appears that the location of the house, which is far from health facilities, will reduce the use of ANC. During the second wave, the difficult situation related to the lockdown policy requires a special strategy for the maternal health sector. This strategy must be able to deal with difficulties in accessing health care facilities during the pandemic. This strategy can be in the form of sending health workers to pregnant women’s homes or through telemedicine. In addition, pregnant women, especially those at high risk, can be mobilized together by ambulance from the Puskesmas to a location not far from people’s homes to go to a referral hospital.

Health facilities that are closed during the pandemic and health facilities that refuse maternal services have no chance of reducing the utilization of maternal services. These results are in line with the results of previous studies [11]. Health facilities that are closed or refuse access do not influence ANC use because the number of these facilities is small compared to health facilities that are open and offer ANC services. Health facilities that are closed are usually because of confirmed COVID-19. Health care facilities that refuse maternal services are usually because the facilities are full; it could be because they allocate most of their services for COVID-19 services, which consumes health facility resources.

What’s interesting about this research is that the most substantial role in utilizing maternal services is the support from husbands and fear during the COVID-19 second-wave attack. These results are in accordance with previous research [11, 19, 20]. This shows the need to increase the husband’s role in the success of antenatal care. In addition, this research shows the need to involve husbands in pregnant classes or pregnant women’s education. Prenatal anxiety, depression, and stress are also considered health problems that often occur among pregnant women [21]. This psychological reaction, in addition to reduced ANC utilization, can also result in miscarriage, premature birth, low birth weight, and fetal death. Overcoming the fear of contracting COVID-19 among pregnant women during this second-wave attack through education regarding prevention procedures, through door-to-door health workers and telemedicine, may overcome this obstacle.

Proper health protocols in health facilities, adequate knowledge of mothers regarding COVID-19, and social media information regarding COVID-19 also seem to increase ANC use during the second-wave attack. These results are in agreement with the research in Ethiopia [11]. Therefore, there is a need for a strict COVID-19 prevention health protocol policy in maternal health services, which must be shared with pregnant women so that they do not hesitate to access maternal health services. Social media information for pregnant women also seems to be a valuable tool in increasing ANC use. It is necessary to encourage pregnant women’s access to information on social media, given that currently, social media is very close to people’s lives, such as WhatsApp groups and YouTube channels.

## Conclusion

Three out of ten pregnant women did not use ANC during the second wave of the COVID-19 pandemic attack. Maternal age, husband’s occupation, distance from the place of residence far from health facilities, poor pregnancy history, husband’s support, the confidence of pregnant women of not contracting COVID-19, easy access to transportation, low ANC costs, absence of a lockdown policy, maternal knowledge regarding COVID-19, information on social media regarding COVID-19, and the use of COVID-19 prevention protocols in health facilities are predictors of ANC utilization. This encourages the importance of prioritizing health services for pregnant women during the pandemic, overcoming the fear of pregnant women contracting COVID-19 through maternal education, husband support, easy access to ANC, and improving the quality of ANC service facilities.

## Acknowledgments

### Conflict of interest

The authors declare no conflict of interest.

### Ethical approval

Ethics approval was obtained from the Ethics Committee of the Bangil Regional Public Hospital of Pasuruan District, Indonesia (approval ID: 445.1/26.424.07201/2021).

### Consent to participate

Written informed consent was obtained from the participants. The research objectives were explained to the participants verbally and in writing, including the rights to refuse or withdraw some or all of their participation in the research. Confidentiality of the subject’s responses was guaranteed throughout the research.
